# Parasite-Mediated Remodeling of the Host Microfilament Cytoskeleton Enables Rapid Egress of Trypanosoma cruzi following Membrane Rupture

**DOI:** 10.1128/mBio.00988-21

**Published:** 2021-06-22

**Authors:** Eden R. Ferreira, Alexis Bonfim-Melo, Barbara A. Burleigh, Jaime A. Costales, Kevin M. Tyler, Renato A. Mortara

**Affiliations:** a Departamento de Microbiologia, Imunologia e Parasitologia, Escola Paulista de Medicina, Universidade Federal de São Paulo, São Paulo, Brazil; b Department of Immunology and Infectious Diseases, Harvard T. H. Chan School of Public Health, Boston, Massachusetts, USA; c Centro de Investigación para la Salud en América Latina, Escuela de Ciencias Biológicas, Pontificia Universidad Católica del Ecuador, Quito, Ecuador; d Biomedical Research Centre, Norwich Medical School, University of East Anglia, Norwich, United Kingdom; e Center of Excellence for Bionanoscience Research, King Abdul Aziz University, Jeddah, Saudi Arabia; University of Georgia

**Keywords:** egress, *Trypanosoma cruzi*, stage-regulated, lytic cycle, microfilament, host-parasite relationship, trypomastigote, amastigote, cell invasion, protozoan, Chagas’ disease

## Abstract

Chagas’ disease arises as a direct consequence of the lytic cycle of Trypanosoma cruzi in the mammalian host. While invasion is well studied for this pathogen, study of egress has been largely neglected. Here, we provide the first description of T. cruzi egress documenting a coordinated mechanism by which T. cruzi engineers its escape from host cells in which it has proliferated and which is essential for maintenance of infection and pathogenesis. Our results indicate that this parasite egress is a sudden event involving coordinated remodeling of host cell cytoskeleton and subsequent rupture of host cell plasma membrane. We document that host cells maintain plasma membrane integrity until immediately prior to parasite release and report the sequential transformation of the host cell’s actin cytoskeleton from normal meshwork in noninfected cells to spheroidal cages—a process initiated shortly after amastigogenesis. Quantification revealed gradual reduction in F-actin over the course of infection, and using cytoskeletal preparations and electron microscopy, we were able to observe disruption of the F-actin proximal to intracellular trypomastigotes. Finally, Western blotting experiments suggest actin degradation driven by parasite proteases, suggesting that degradation of cytoskeleton is a principal component controlling the initiation of egress. Our results provide the first description of the cellular mechanism that regulates the lytic component of the T. cruzi lytic cycle. We show graphically how it is possible to preserve the envelope of host cell plasma membrane during intracellular proliferation of the parasite and how, in cells packed with amastigotes, differentiation into trypomastigotes may trigger sudden egress.

## INTRODUCTION

The protozoan parasite Trypanosoma cruzi is the etiological agent of Chagas’ disease, a deadly and vector-borne zoonotic disease of poverty that affects 6 to 7 million people, mostly in South and Central America, and which lacks vaccines and effective therapeutics (1). During its lytic cycle in the mammalian host, T. cruzi alternates between extracellular and infective trypomastigotes and intracellular proliferative amastigotes (2, 3). The lytic cycle was first filmed by Hertha Meyer in the 1940s (4) and is canonically described as attachment to the host cell, vacuolar escape, replication of the amastigote in the cytosol, and lytic egress. Studies on the early part of the lytic cycle have yielded considerable insight into fundamental cellular mechanisms, such as wound healing and lysosomal exocytosis. The initial signaling cascades associated with cell invasion and cytoskeletal remodeling and the mechanisms of vacuolar biogenesis and escape are well described (5–11). In comparison, little is understood about the latter stages of the lytic cycle: the parasite’s exit strategy and how the environment provided by the host cell is modified by T. cruzi to facilitate its release. It has, though, been proposed that the movement of the trypomastigote forms may lead to mechanical rupture of the host cell (12) and that surface expression or secretion of proteases may contribute to cytoskeleton and membrane disruption (13–15).

Using live-cell imaging and correlative microscopy, we provide a detailed temporal and morphological dissection of the latter part of the lytic cycle leading to T. cruzi egress. We demonstrate egress as a well-orchestrated event involving substantial actin cytoskeleton remodeling to accommodate intracellular proliferation and where microfilament barrier degradation with the participation of T. cruzi protease and torsion from the motile trypomastigote forms deliver the appropriately timed triggers for subsequent egress. In contrast, our results do not provide evidence of gradual membrane disintegration marked by attendant changes in membrane permeability but instead support the sudden egress of trypomastigotes following membrane rupture.

## RESULTS

### Trypanosoma cruzi egress is a sudden event with no membrane perturbation prior to parasite egress.

**FIG 1:**
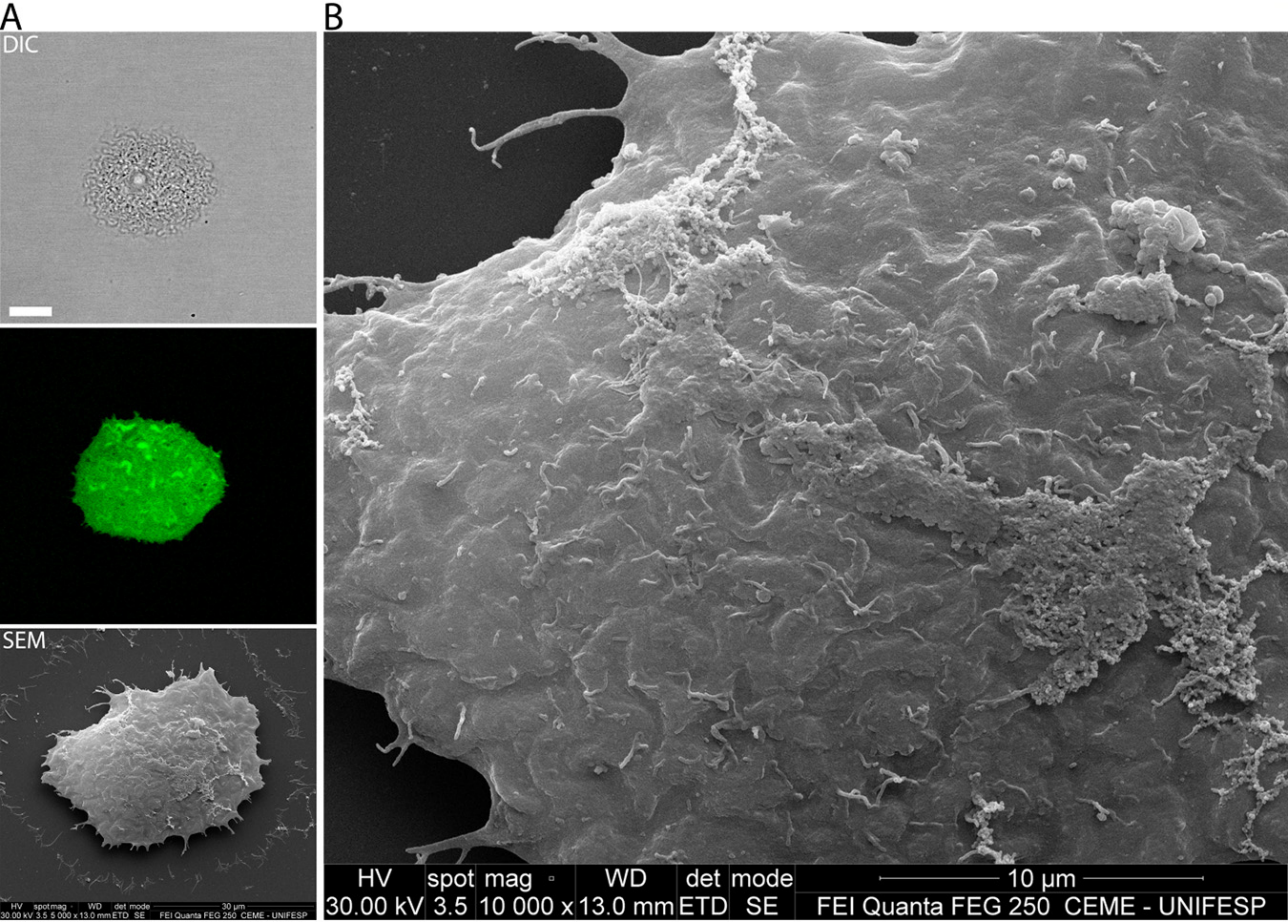
No host cell plasma membrane degradation is observed immediately prior to egress. Cells were first observed by confocal microscopy and later imaged by SEM. (A, upper panel) DIC. Size bar, 15 μm. (Middle panel) The CMFDA marker shows that there is no cytoplasmic content leaking prior to egress. (Lower panel) Same cell observed by SEM (correlative microscopy). (B) Enlarged portion of the same cell, showing no ruptures or pores at the membrane. Size bar, 10 μm. In this image, it is possible to observe the trypomastigotes in low relief beneath the host cell plasma membrane.

In order to directly observe membrane integrity of cells prior to trypomastigote release, we performed correlative microscopy: first monitoring cells in late stages of the lytic cycle by confocal live-cell imaging and then visualizing the same cell by scanning electron microscopy (SEM) (Fig. 1). CMFDA (5-chloromethylfluorescein diacetate) is a vital marker that is cell membrane permeable and nonfluorescent. In the cytosol, CMFDA is cleaved by cytoplasmic esterases and becomes membrane impermeant and fluorescent (16). Direct observation of cells stained with CMFDA affirms the integrity of the plasma membrane, showing no leakage of the cytosolic marker prior to parasite egress (Fig. 1A), while SEM of the same cells showed no visible evidence of membrane damage or disruption (Fig. 1B). Video S1 in the supplemental material shows the intense movement of trypomastigotes inside the host cell characteristic of the late lytic cycle immediately prior to egress.

**FIG 2:**
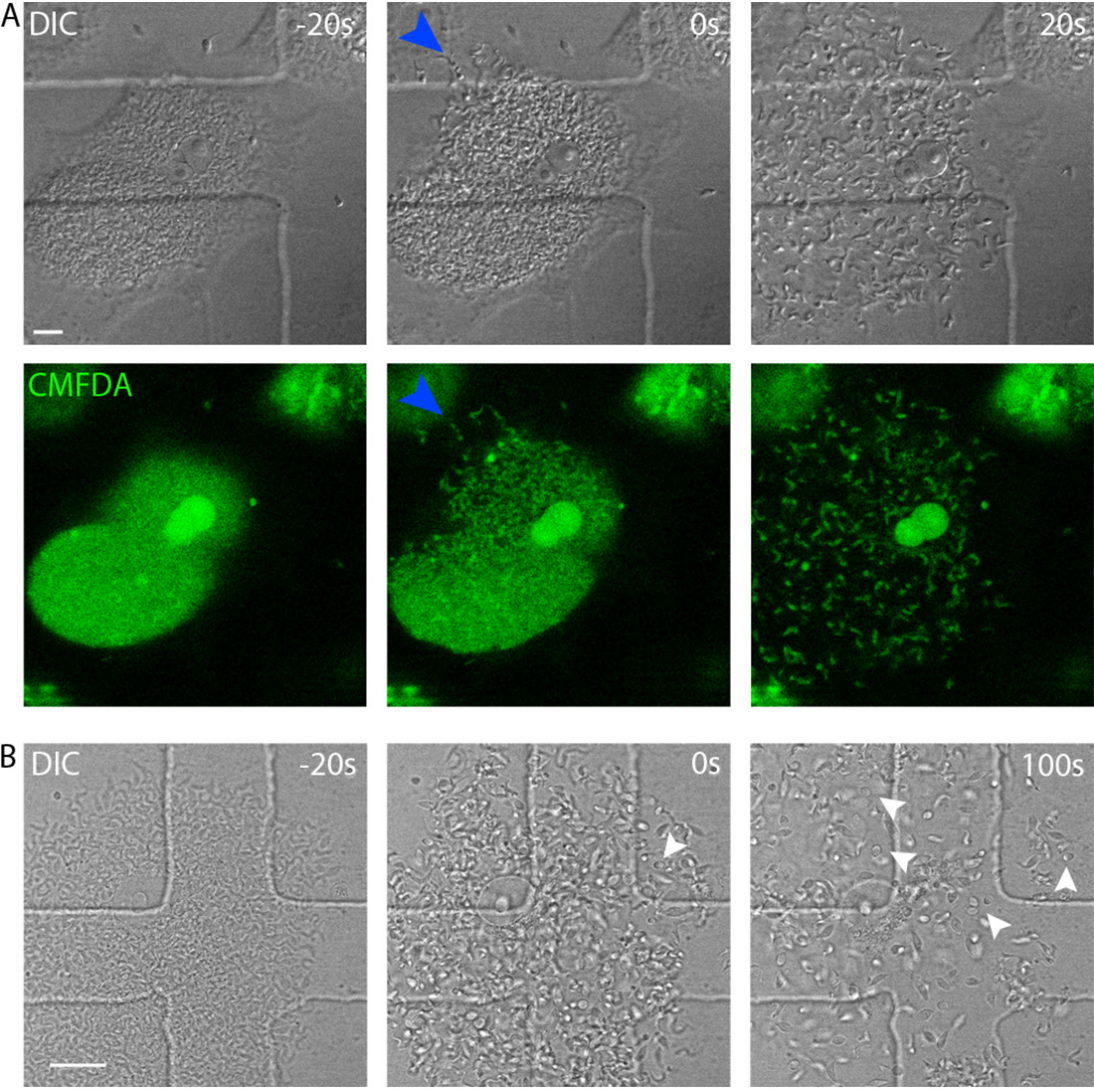
T. cruzi egress is a rapid event. (A) Vero cells, infected with T. cruzi trypomastigote forms, were visualized by confocal live-cell imaging up to parasite egress. −20 s, pre-egress; 0 s, moment of egress; 20 s, after egress. Images were acquired with a capture interval of 20 s. Cells were preincubated with CMFDA. Blue arrowheads indicate the site of membrane rupture. The image is representative of 80 observed events. Size bar, 10 μm. (B) Amastigotes can be released during T. cruzi egress. Arrowheads point to some of the released amastigotes, and several other amastigotes can be found in the field of view. −20 s, pre-egress time point; 0 s, moment of egress; 100 s, time point that better shows released amastigotes. Time interval between frames, 20 s. Size bar, 20 μm.

We also evaluated whether T. cruzi egress would be a slow process with gradual liberation of trypomastigotes or an abrupt lytic event. As shown in Fig. 2 and Video S2 in the supplemental material, T. cruzi egress seems to be a rapid event, with sudden host cell membrane rupture and instant parasite release. CMFDA stain highlights the precise moment of rupture as it engenders a sudden fluorescence loss when the cytosolic compartment is breached. This is also indicated by abrupt although discrete change in cell refringence (Fig. 2, differential inference contrast [DIC] image at 0 s compared with −20 s).

We observed egress by this method from a large number of infected cells (*n* = 80). Interestingly, although in all cases at least some trypomastigotes egressed from the cell, other morphologies were sometimes also present. In Fig. 2B, we show amastigotes and intermediate forms were also released to the extracellular medium.

**FIG 3:**
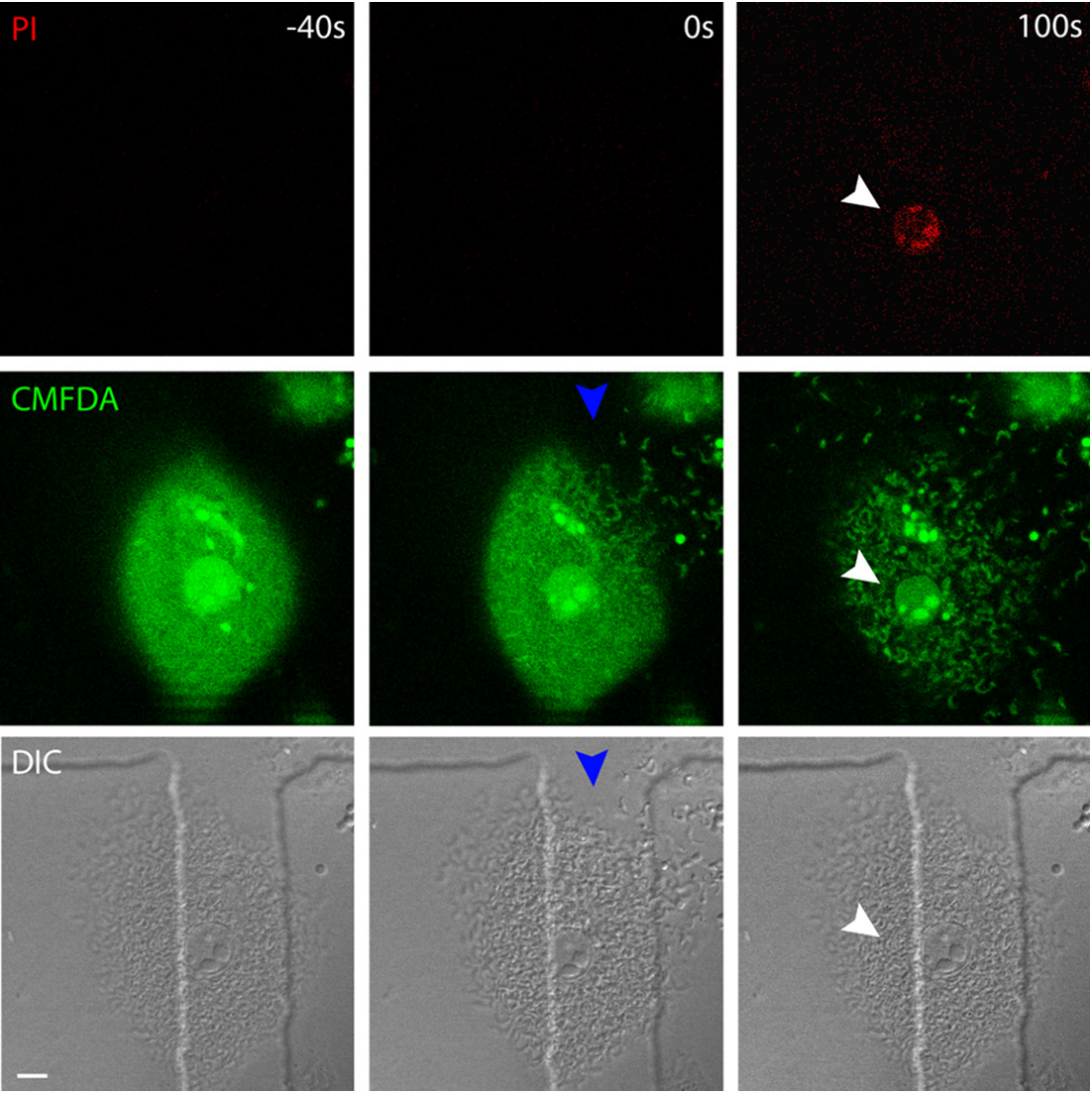
Infected cells stain for propidium iodide only after parasite egress. Infected cells were submitted to confocal live-cell imaging and monitored up to parasite egress. The image shows that prior to egress (−40 s), there is no propidium iodide stain of the nucleus. We observed PI staining of the nucleus only 100 s after parasite egress. The image is representative of 60 observed cells. CMFDA was used to monitor membrane rupture. White arrowheads indicate the nucleus. As indicated by blue arrowheads, it is possible to observe the site of membrane rupture. Size bar, 10 μm.

We also evaluated whether cells immediately prior to egress would show greater permeability to propidium iodide (PI). In order to answer that question, we added PI to the cell culture medium and observed the cells by confocal live-cell imaging. (PI is fluorescent when intercalated with nucleic acids.) Our experiments showed that cells do not stain with PI prior to egress, confirming our results with CMFDA that host cells maintain membrane integrity until the rupture (Fig. 3; see Video S3 in the supplemental material).

### Remodeling of the host microfilament cytoskeleton during the lytic cycle.

**FIG 4:**
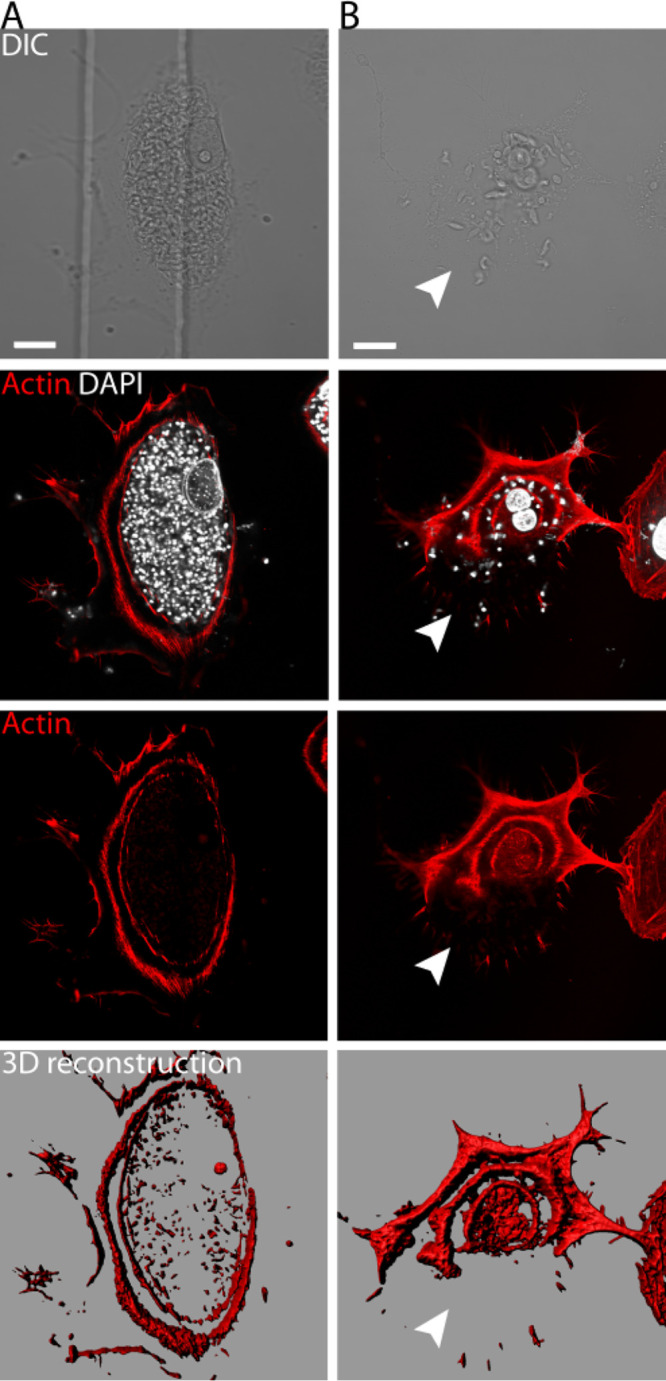
Actin cage-shaped structures observed in cells before and after parasite egress. (A) Vero cells infected with trypomastigotes prior to egress show F-actin cages. Images were obtained by confocal microscopy. A three-dimensional (3D) topological rendering of the actin cytoskeleton provides a detailed model of the actin cages observed (see Videos S5 and S6 in the supplemental material). (B) Infected Vero cells monitored by live-cell imaging up to trypomastigote egress were fixed and incubated with phalloidin-TRITC. (To observe the same cell after fixing, we used a grid coverslip [see Fig. 5 and Video S4].) Three-dimensional topological micrographs of the actin structures were generated using Imaris software (Surface tool). Size bar, 15 μm. Arrowheads indicate the entire portion of membrane rupture, seen also in Fig. 5 and Video S4.

Cortical microfilaments underlie the plasmalemma and can act to protect and restrict access to the plasma membrane. Since we found that plasma membrane integrity is preserved until rupture, we considered whether a microfilament barrier might be serving to contain parasites within the cytosol, thereby preserving plasma membrane integrity prior to egress. We investigated this possibility using phalloidin-tetramethyl rhodamine isothiocyanate (TRITC) on a population of infected cells. For cells late in the lytic cycle, we were surprised by the striking observation of spheroid actin cages surrounding the parasites instead of a normal actin meshwork. Figure 4A shows an infected cell with trypomastigotes prior to egress and compares it to a cell fixed immediately after parasite egress (Fig. 4B; see also Videos S4, S5, and S6 in the supplemental material). It is noteworthy that at the membrane rupture site, part of the cage structure is absent (Fig. 4B; see Video S4 showing the same cell in live-cell imaging).

10.1128/mBio.00988-21.3 VIDEO S1 Vero cells infected with highly motile trypomastigotes. Download VIDEO S1, AVI file, 14.4 MB. Copyright © 2021 Ferreira et al. 2021 Ferreira et al. https://creativecommons.org/licenses/by/4.0/ This content is distributed under the terms of the Creative Commons Attribution 4.0 International license.

10.1128/mBio.00988-21.4 VIDEO S2 Live-cell imaging of the Vero cell until parasite egress. Download VIDEO S2, AVI file, 12.7 MB. Copyright © 2021 Ferreira et al. 2021 Ferreira et al. https://creativecommons.org/licenses/by/4.0/ This content is distributed under the terms of the Creative Commons Attribution 4.0 International license.

10.1128/mBio.00988-21.5 VIDEO S3 Propidium iodide stain shows that normal host cell membrane permeability is maintained until parasite egress. Download VIDEO S3, AVI file, 15.2 MB. Copyright © 2021 Ferreira et al. 2021 Ferreira et al. https://creativecommons.org/licenses/by/4.0/ This content is distributed under the terms of the Creative Commons Attribution 4.0 International license.

10.1128/mBio.00988-21.6 VIDEO S4 Vero cells infected with trypomastigotes observed up to parasite egress. Download VIDEO S4, AVI file, 16.4 MB. Copyright © 2021 Ferreira et al. 2021 Ferreira et al. https://creativecommons.org/licenses/by/4.0/ This content is distributed under the terms of the Creative Commons Attribution 4.0 International license.

10.1128/mBio.00988-21.7 VIDEO S5 Three-dimensional surface showing actin cages formed in a cell infected with trypomastigotes. Download VIDEO S5, AVI file, 12.7 MB. Copyright © 2021 Ferreira et al. 2021 Ferreira et al. https://creativecommons.org/licenses/by/4.0/ This content is distributed under the terms of the Creative Commons Attribution 4.0 International license.

10.1128/mBio.00988-21.8 VIDEO S6 Three-dimensional surface showing an infected cell after parasite egress and the actin cages formed due to infection. Download VIDEO S6, AVI file, 12.2 MB. Copyright © 2021 Ferreira et al. 2021 Ferreira et al. https://creativecommons.org/licenses/by/4.0/ This content is distributed under the terms of the Creative Commons Attribution 4.0 International license.

**FIG 5:**
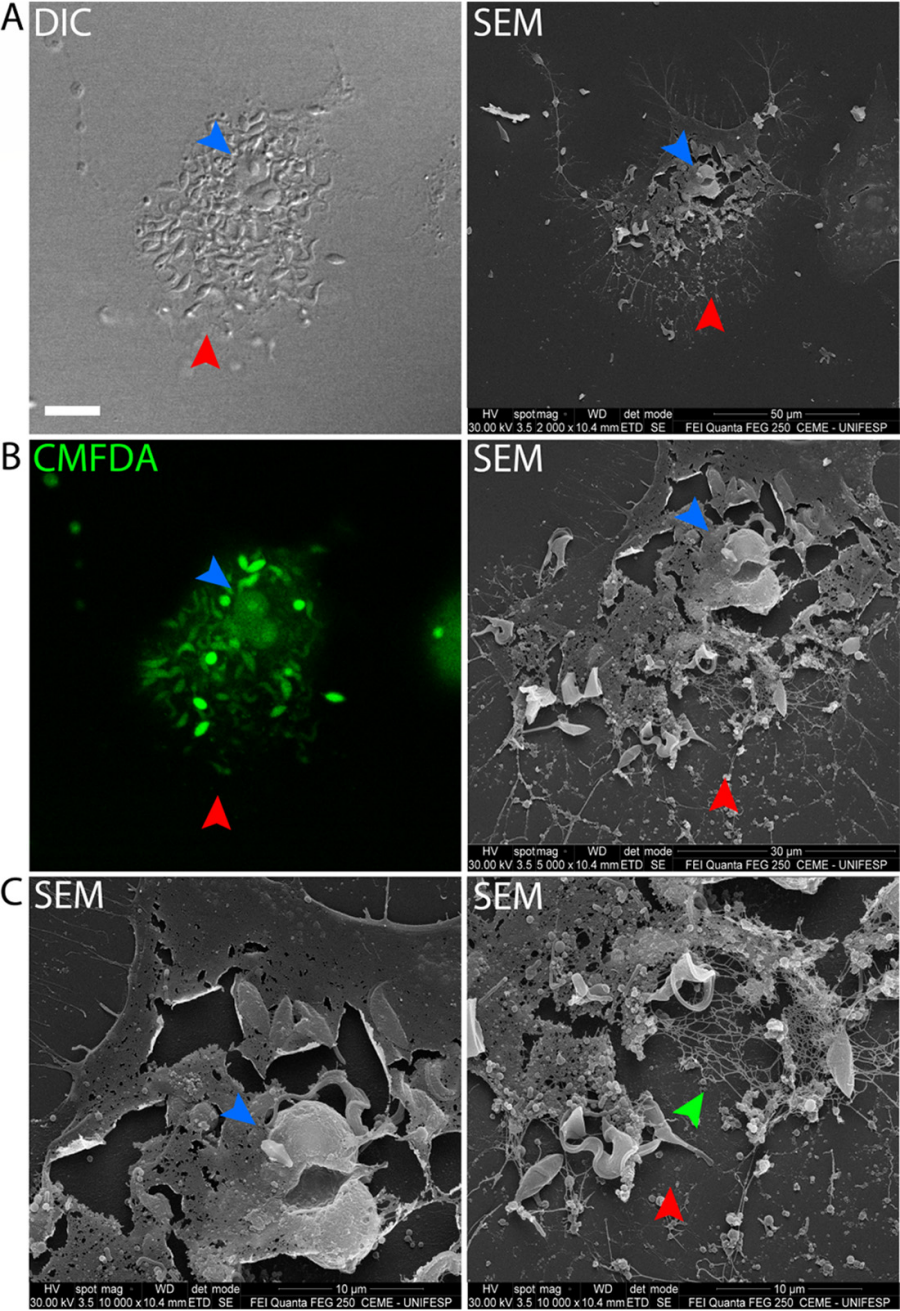
Host cell remains after parasite egress. (A) DIC and SEM showing correlative images of the same cell at the moment of parasite egress (DIC; size bar, 15 μm). (B, left panel) CMFDA staining used to assist egress precise moment. (Right panel) Magnified SEM image of the host cell. (C, right and left panels) SEM higher magnification. It is possible to observe the remains of the host cell cytoskeleton (green arrowhead) and nucleus (blue arrowhead). The red arrowhead maps to the membrane rupture region.

The infected cell shown in Fig. 4B was also prepared for SEM (correlative microscopy). This additional resolution shows the absence of filamentous cytoskeleton at the actual rupture site but retention of at least some filamentous cytoskeletal elements peripheral to the rupture site and elsewhere in the cell (Fig. 5; Video S4). Together, Fig. 4 and 5 support the notion of an abrupt egress following a break in the filamentous actin cytoskeleton and subsequent egress through the proximate plasma membrane.

**FIG 6:**
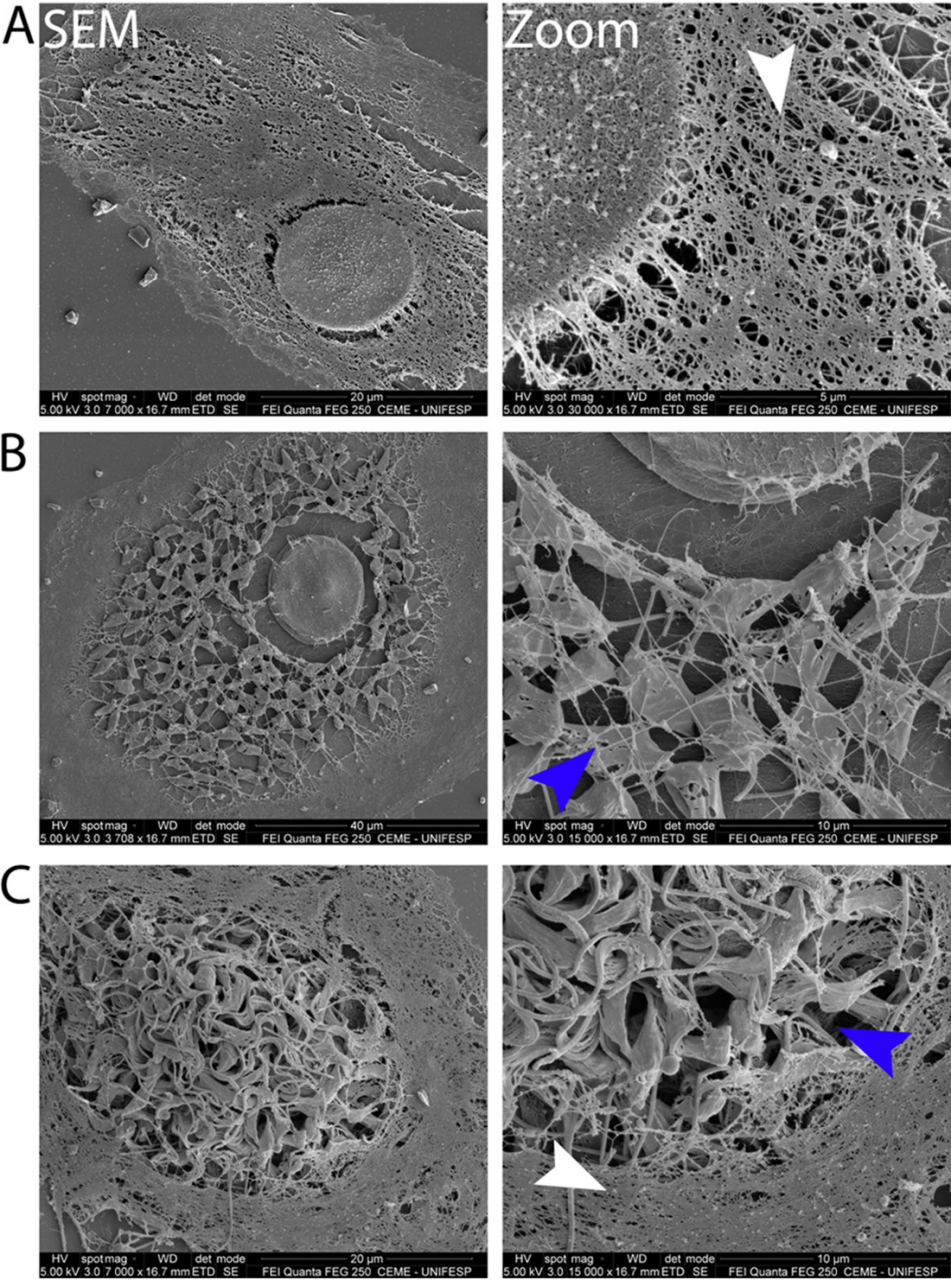
Cytoskeleton is gradually disrupted during parasite infection. Shown are scanning electron microscopy (SEM) images of Vero cells treated with Triton X-100-based solution containing phalloidin and paclitaxel (to stabilize cytoskeleton filaments). Images represent the gradual cytoskeleton disruption during the parasite lytic cycle. (A) Noninfected cell with dense meshwork of cytoskeleton. (B) Infected cell at the amastigote stage showing a less dense meshwork. (C) Infected cell with parasites at the trypomastigote stage showing the lack of an overlaying meshwork. It is possible to visualize actin filaments forming a peripheral cage surrounding the trypomastigotes. Zoom images (right panels) show loss of cytoskeleton of infected cells. Blue arrowheads show parasite sites with less or no cytoskeleton, and white arrowheads show preserved cytoskeleton filaments.

**FIG 7:**
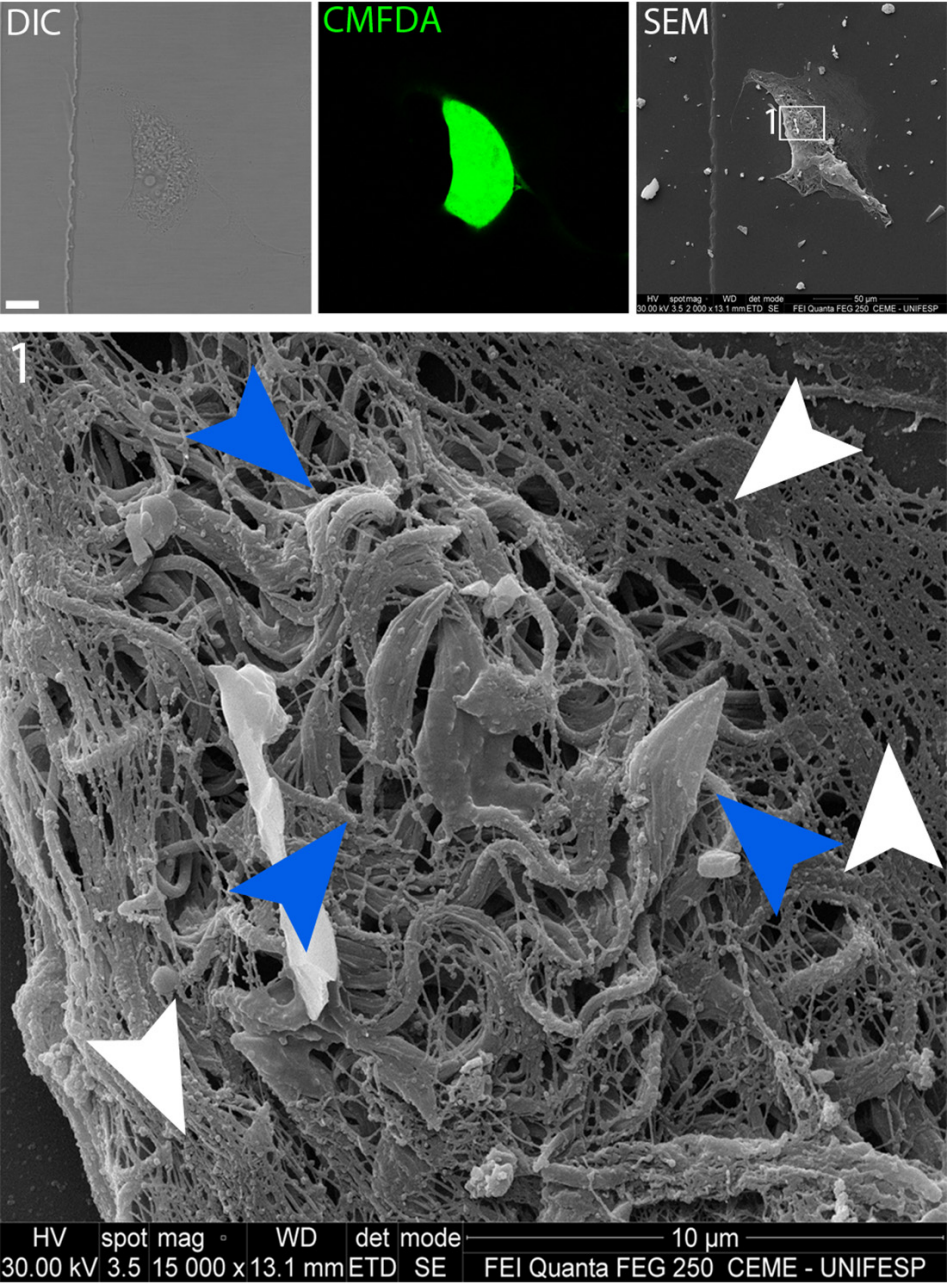
Cytoskeleton disruption at intracellular trypomastigote sites. The SEM image shows that in cells infected with T. cruzi at the trypomastigote stage, it is still possible to see cytoskeleton filaments (white arrowheads). At the trypomastigote site (blue arrowheads), we observe less or no cytoskeleton structures.

Since we observed a partial disruption of the cytoskeleton, we considered more broadly the possible cytoskeleton alterations due to T. cruzi infection. In order to do so, we used 1% Triton X-100 at room temperature to demembranate infected cells, stabilized with paclitaxel (originally named taxol) and phalloidin, and stained microfilaments with fluorescently labeled phalloidin (17, 18). After detergent treatment, cells were fixed and observed by SEM. Corroborating our fluorescence microscopy results, SEM showed that T. cruzi disrupts host cell cytoskeleton during its intracellular development, resulting in a progressively lower density of filaments over the course of the lytic cycle (Fig. 6). In comparison with noninfected cells (Fig. 6A), cytoskeleton disruption in parasitized cells becomes evident after vacuolar escape and once amastigote proliferation is well under way, and amastigotes appear to be overlaid by a loose mesh of filaments (Fig. 6B). Once trypomastigotes are formed, the density of microfilaments falls further still, and there is an almost complete loss of discernible cytoskeletal filaments closely associated with or overlaying the trypomastigotes in some cells, particularly those where density of trypomastigotes is high (Fig. 6C). Corroborating Fig. 6, Fig. 7 shows correlative microscopy demonstrating that despite the cell membrane integrity seen by CMFDA stain, host cell cytoskeleton is disrupted at the late stages of infection.

**FIG 8:**
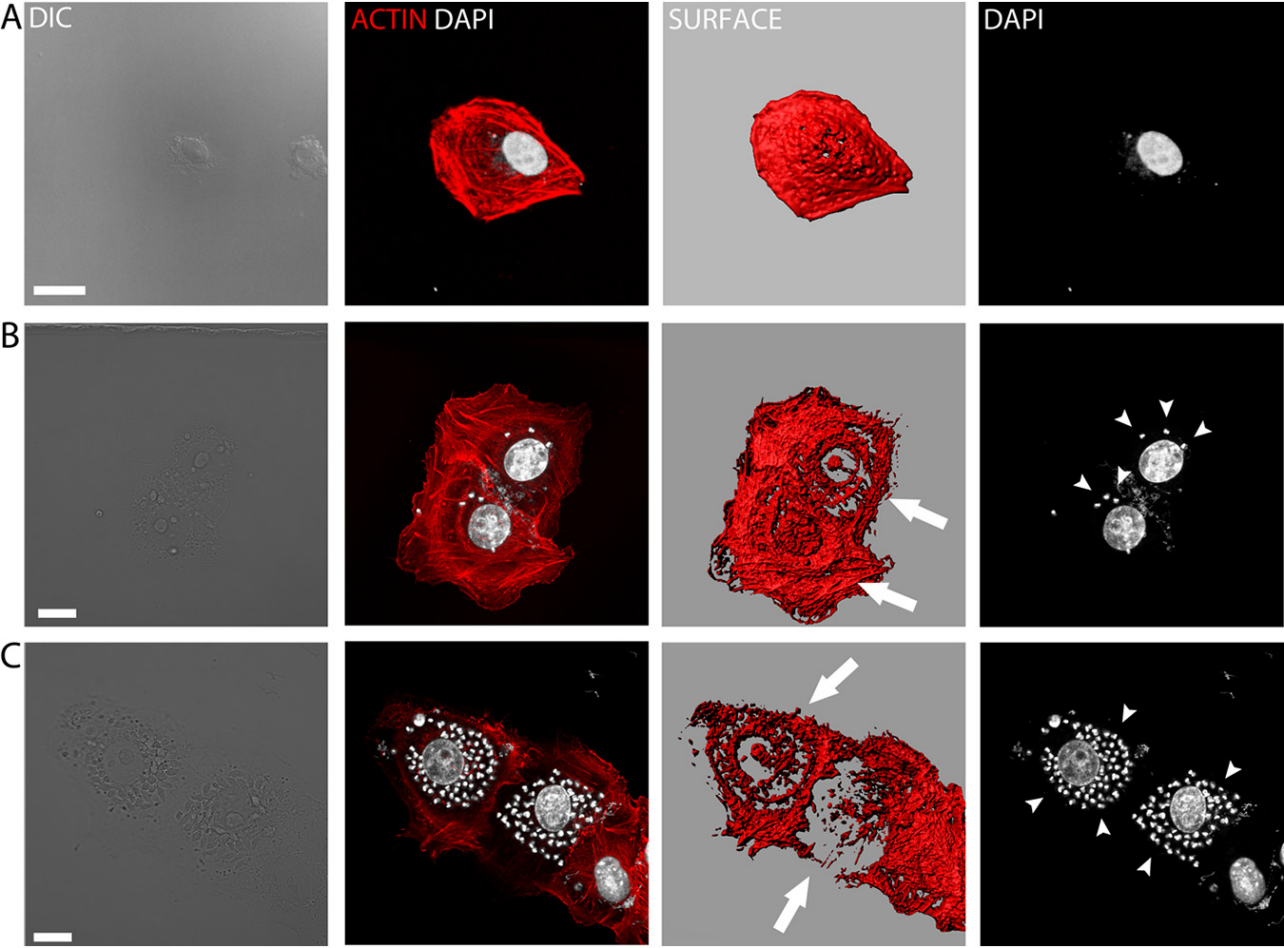
Actin cage structures are formed in cells infected with amastigotes. Infected Vero cells during the intracellular amastigote stage were fixed and incubated with phalloidin-TRITC for actin cytoskeleton observations. (A) Noninfected cell. (B and C) Cells containing amastigotes (arrowheads). Actin cages are clearly visible (Arrows). Images were obtained by confocal microscopy and reconstructed using Imaris software. Size bar, 15 μm.

**FIG 9:**
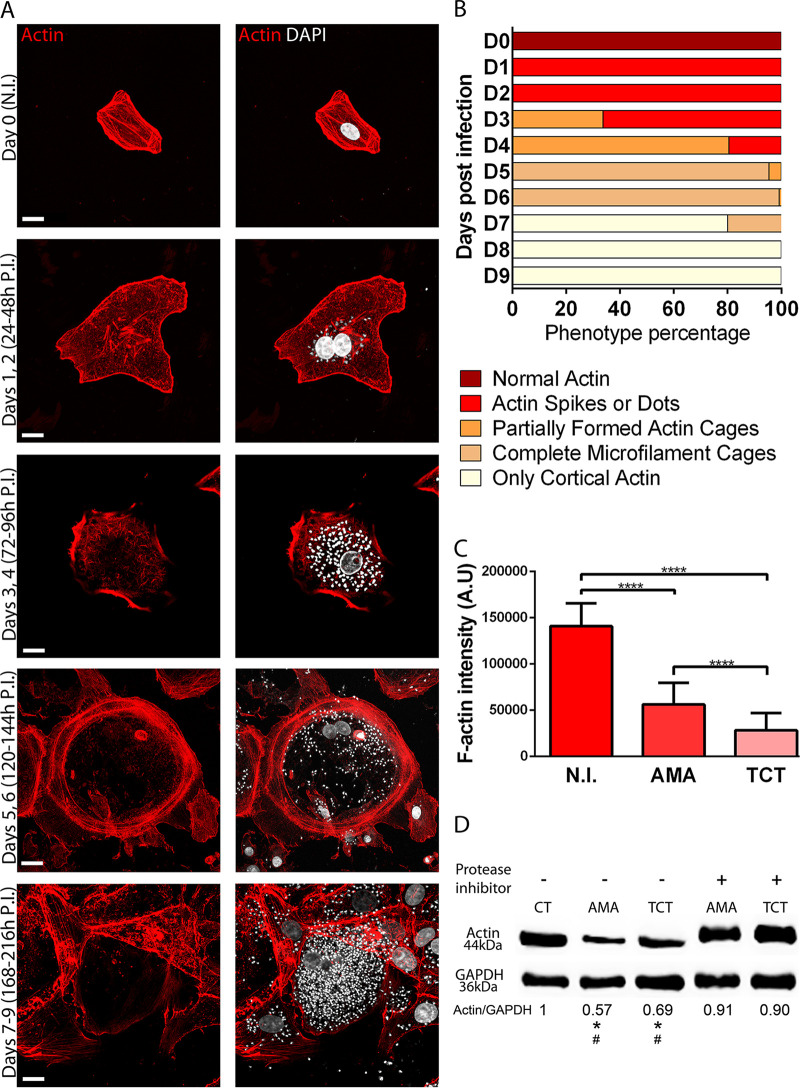
T. cruzi intracellular development promotes actin cytoskeleton disassembly, likely to be mediated by parasite proteases. (A to C) Actin cytoskeleton changes from a dense actin meshwork to almost cortical actin only in late stages of the T. cruzi intracellular cycle. (D) T. cruzi amastigote- and trypomastigote-expressed proteases participate in degradation of host cell actin cytoskeleton. (A) Infected cells were fixed and stained with phalloidin-TRITC, and actin patterns were observed and photographically recorded each day. Representative images of an infected cell from each day are shown. On day 0, there is no infection (N.I.). At 24 to 48 h postinfection (P.I.), there is a dense actin meshwork. At 72 to 96 h postinfection, actin spikes appear on the cytoplasm. At 120 to 144 h postinfection, there is spheroidal actin cage formation. By days 7 to 9 (168 to 216 h postinfection), actin cytoskeleton cages are completely formed. Most of the actin stress fibers are absent. Size bar, 15 μm. (B) Actin morphologies of infected cells were evaluated under epifluorescence microscopy during intracellular parasite development. To precisely determine the actin cytoskeleton pattern during infection, two independent experiments were performed, and three coverslips per group were counted, at 100 cells per coverslip. (C) Measurements of phalloidin-TRITC intensity show F-actin loss in cells infected with amastigotes (AMA) and trypomastigotes (TCT) compared with noninfected cells (N.I.). Measurements were performed on ImageJ using the Measurement tool. Signal intensity is represented by arbitrary units (A.U.). The results represent the mean + standard deviation (SD) from two independent experiments in triplicate (one-way ANOVA; ****, *P* < 0.0001). (D) Vero cell lysates were incubated for 3 h at 37°C, indicated as follows: CT, cell lysates without parasite; AMA and TCT, cell lysates incubated with amastigotes and trypomastigotes, respectively. “−” and “+” indicate the absence or presence of protease inhibitor cocktail, respectively. Anti-GAPDH was used as loading control. Significant (*P* < 0.05) reductions of actin signal from AMA and TCT groups compared with the control group (*) or compared with TCT and AMA treated with protease inhibitors (#) are indicated. The image is representative of three independent experiments, and statistical analysis was performed by one-way ANOVA.

To further investigate the apparent degradation of microfilament cytoskeleton during the lytic cycle, we first stained infected cells with phalloidin-TRITC. Confocal microcopy images confirmed our impression that actin cytoskeleton changes first become detectable after vacuolar escape and amastigogenesis once amastigote proliferation is under way (Fig. 8). We undertook a time course experiment evaluating change to the host actin cytoskeleton during the lytic cycle, monitoring host cells daily from infection on day 0 to day 9—when most of the cells are close to, or just prior to, egress (Fig. 9A and B). Again, the fluorescence microscopy study confirmed our impression from SEM of a significant reduction in filamentous actin (phalloidin-TRITC), first in cells infected with amastigotes (days 3 to 5) and then more dramatically in cells containing trypomastigotes (days 6 to 9). Figure 9C shows a decrease in F-actin signal (phalloidin-TRITC) in cells infected with amastigotes and trypomastigotes. In line with this observation, Western blotting experiments showed host cell actin degradation by T. cruzi is likely to be driven by parasite protease activity. Figure 9D indicates that incubation of cell lysates with amastigotes or trypomastigotes reduces actin signal, which is avoided when cell lysates are incubated with parasites in the presence of protease inhibitors.

Our observations were also extended to a different cell model in order to investigate the universality of T. cruzi egress. Using HeLa cells infected with the T. cruzi Y strain, very similar results to those from Vero cells infected with the G strain were observed. Figure S1A and Video S7 in the supplemental material demonstrate the sudden egress of trypomastigotes from HeLa cells. Figure S1B shows the loss of actin cytoskeleton in cells infected with trypomastigotes (lower right side) in comparison and side by side with noninfected cells (upper left side). In addition, Western blotting experiments revealed confirmed loss throughout the days of infection in HeLa cells (Fig. S1C).

10.1128/mBio.00988-21.1 FIG S1 HeLa cells infected with the Y strain display similar egress features to Vero cells infected with the G strain. (A, upper panel) PI stains the nucleus only after parasite egress. (Middle panel) Cytoplasmic CFMDA stain is instantly lost in the moment of parasite egress. (Lower panel) DIC images showing parasite egress. White arrowheads indicate host cell nuclei. HeLa cells infected with T. cruzi trypomastigote forms were visualized by confocal live-cell imaging up to parasite egress. Size bar, 10 μm. (B) Actin cytoskeleton of HeLa cells is reduced in late stages of infection (trypomastigotes). The upper left portion shows cells with low or no infection (white arrowheads). The lower right portion shows cells heavily infected with trypomastigotes (seen with DAPI stain). Images were obtained by confocal microscopy and reconstructed using Imaris software. Size bar, 20 μm. (C) Western blotting experiments using anti-β-actin show progressive reduction of β-actin in HeLa cells infected with the Y strain. N.I., noninfected cells as a control; D3, third day of infection; D5, fifth day of infection; D7, seventh day after infection. Coomassie membrane stain was used to confirm equal amounts of loaded cell lysate. Download FIG S1, JPG file, 1.6 MB. Copyright © 2021 Ferreira et al. 2021 Ferreira et al. https://creativecommons.org/licenses/by/4.0/ This content is distributed under the terms of the Creative Commons Attribution 4.0 International license.

Furthermore, immunofluorescence experiments using anti-β-actin demonstrated that for both HeLa and Vero cells, total actin signal is reduced in infected cells compared to noninfected cells (see Fig. S2 in the supplemental material). The reduction of actin fluorescence signal in infected cells is clearly observed. Together, these results demonstrate the universality of T. cruzi egress and also indicate that intracellular development of T. cruzi induces host cell G- and F-actin modifications.

10.1128/mBio.00988-21.2 FIG S2 Immunofluorescence using anti-β-actin reveals total actin (G- and F-actin) reduction in cells infected with T. cruzi. (A) Vero cells. (B) HeLa cells. N.I., AMA, and TCT indicate noninfected cells and cells infected with amastigotes and trypomastigotes, respectively. Arrowheads indicate the reduction in total actin, detected with anti-β-actin (green). Phalloidin-TRITC was used to stain F-actin and DAPI to stain the nucleus and kinetoplast. Images were obtained by confocal microscopy and reconstructed using Imaris software. Size bar, 30 μm. Download FIG S2, JPG file, 2.3 MB. Copyright © 2021 Ferreira et al. 2021 Ferreira et al. https://creativecommons.org/licenses/by/4.0/ This content is distributed under the terms of the Creative Commons Attribution 4.0 International license.

## DISCUSSION

The lytic cycle of T. cruzi (attachment, invasion, proliferation, differentiation, and egress) and its central role in Chagas’ disease pathogenesis have been known for over a century. Disruption of any aspect of the lytic cycle is a valid therapeutic target in a disease for which new targets are still much needed. However, while our understanding of how the host cell becomes parasitized has made great strides and provided us with increased understanding of calcium-mediated exocytosis, lysosomal trafficking, wound healing, and entosis (5, 7–9, 19–21), very little has been gleaned about the other equally critical end of the lytic cycle—the parasite’s highly successful and highly coordinated cellular escape strategy. We provide here the first substantial article to address the cellular mechanism of T. cruzi egress.

We initially considered the kinetics of parasite egress and whether egress followed a lytic event as it does in other intracellular parasitism, such as malaria and *Toxoplasma* infection (22–24), or whether parasites were being released more slowly over a protracted period while the cell remained intact. Live-cell imaging of the T. cruzi lytic cycle was initially recorded in the 1940s by Hertha Meyer (4). A movie displays the dramatic moment when the trypomastigotes break through the host cell membrane and parasites are rapidly released. Our results bring more detail to those seminal observations, and in our experiments, cells were incubated with CMFDA and PI in order to monitor the plasma membrane breach attendant with egress. Our correlative SEM experiments confirmed results from live-cell imaging and showed that in cells containing highly motile trypomastigotes, there was no evidence of pore formation or other forms of membrane breach prior to parasite egress. These findings support the notion that during its intracellular life cycle the parasites manage to prepare the cell for egress only when most of intracellular parasites are fully formed into trypomastigotes ready to exit and colonize other cells.

Our results showed that there is no PI nuclear staining or CMFDA fluorescence loss from infected cells prior to egress, demonstrating maintenance of host cell membrane integrity up to rapid release. However, Costales and Rowland (13) suggested that the membrane of some infected cells may become permeable to trypan blue prior to egress and that paraformaldehyde-fixed but not permeabilized cells can be permissive to penetration of amastigote-specific antibodies. In order to clarify those results, we present here similarly confluent cell monolayers, but at higher magnification to observe the moment of egress. Video S8 in the supplemental material illustrates that in confluent monolayers, it is difficult to determine the precise moment of host cell plasma membrane rupture prior to parasite egress, since the tight packing of cells delays trypomastigote dispersion. Only by using time-lapse imaging plus CMFDA and PI staining under high magnification could we determine the precise moment of membrane rupture. Regarding paraformaldehyde fixation, as suggested by the authors, some membrane weakening might occur at later stages of infection, making the membrane permeable after fixation. We could speculate that alive infected cells might continuously heal the membrane and would thus appear impermeable in our assays, while this would cease after fixing, which would reconcile the results described by Costales and Rowland, who observed antibody penetration through the membrane in mammalian cells containing amastigotes on day 3 of infection, which is early for a membrane breach prior to egress. Moreover, recent studies have also revealed that paraformaldehyde fixation solubilizes membrane lipids compromising membrane integrity (25, 26). Therefore, taken together with the new data presented, the Costales and Rowland observations may be attesting to a difference in plasma membrane composition late in the lytic cycle and prior to egress rather than a difference in membrane integrity per se, and indeed this certainly warrants further investigation in the future.

Our results also demonstrated that actin cytoskeleton is gradually rearranged during parasite intracellular development and that over the course of the lytic cycle, the level of actin in general and filamentous actin in particular declines. From the first day after infection, we observed differences to the actin cytoskeleton pattern from normal stress fibers, to actin cages during amastigote multiplication and differentiation into trypomastigotes, and to an absence of stress fibers in cells just prior to egress and containing highly motile trypomastigotes. Low et al. (15) have previously suggested that protease activity may be associated with egress. Our results support that contention; we showed that cell lysates incubated with amastigotes or trypomastigotes mediate a clear reduction in the host actin present in host cell lysates, analogous to the observed loss of actin during the course of the lytic cycle, and this degradation of host actin is rescued by the presence of protease inhibitors. Taken together with our observations of an overall loss of filamentous actin overlaying intracellular trypomastigotes and the known presence of powerful surface proteases (17, 27, 28), it is possible to surmise that it is the accumulation of parasite protease over the lytic cycle that drives degradation of the actin barrier until it is sufficiently weakened that motility of the trypomastigote forms can provide sufficient torsion to breach and then rupture the membrane, which triggers subsequent rapid egress.

For other intracellular protozoan parasites—*Leishmania* spp., *Plasmodium* spp., and Toxoplasma gondii—parasite protease activity is also intimately associated with egress (24, 29). However, other cytoskeleton-modulating mechanisms have also been invoked for egress by other parasites. For the protozoan parasite *Plasmodium*, it is known that while in red blood cells, the parasite induces the loss of cytoskeleton adaptor proteins, including α/β-adducin and tropomyosin, correlating temporally with the emergence of large gaps in the cytoskeleton (30). Similar results were found for Toxoplasma gondii, and the authors believe that parasites first remove a selected set of cytoskeletal adaptor proteins to weaken the host membrane and then use host calpain-1 to dismantle the remaining cytoskeleton, leading to host cell membrane collapse and parasite release (23, 30). Therefore, while protease-mediated actin degradation is likely to be a key factor triggering Trypanosoma cruzi egress, we cannot exclude roles for other mechanisms by which the filamentous actin may be denuded, and moreover, we have not ruled out a role for other elements of the cytoskeleton. For instance, Mott et al. (14) demonstrated that actin filaments might be weakened during the intracellular amastigote stage due to protein kinase A (PKA) activation and Rho/Rho kinase pathway inhibition. Moreover, it has been suggested that vimentin and myofibrils (in cardiomyocytes) (31) might be weakened by intracellular infection. For vimentin, it has been described that during amastigote differentiation into trypomastigotes, the parasite secretes a phosphoinositide phospholipase C (PI-PLC) enzyme that is believed to hydrolyze phosphatidylinositol 4,5-bisphosphate (PIP_2_), consequently destabilizing the vimentin-based intermediate filaments (32). In sum, our results provide the first description of the cellular mechanism that regulates the lytic component of the T. cruzi lytic cycle. We show graphically how it is possible to preserve the envelope of the host cell plasma membrane during intracellular proliferation of the parasite and how in a cell packed with amastigotes, differentiation into trypomastigotes may trigger sudden egress. These studies open the way to a molecular dissection of the associated pathways and cellular infrastructure underpinning egress by Trypanosoma cruzi that should be addressed in future studies.

## MATERIALS AND METHODS

### Parasites and cells.

In this study, we used tissue culture trypomastigotes (analogous to bloodstream trypomastigotes) from G and Y strains (33, 34): T. cruzi I and II, respectively. Trypomastigotes were obtained by collection of culture medium from infected Vero cells (epithelial cells extracted from an African green monkey [Chlorocebus sabaeus]) on the sixth or seventh day after cell infection. HeLa cells (human cervical adenocarcinoma cells) and Vero cells were used for experiments of infection and egress. Those cells were cultivated in RPMI 1640 medium (Sigma-Aldrich) supplemented with 10% fetal bovine serum (FBS; Invitrogen), 10 μg ml^−1^ streptomycin, 100 U ml^−1^ penicillin, and 40 μg ml^−1^ gentamicin at 37°C and 5% CO_2_ in RPMI–10% fetal calf serum.

### Host cell membrane permeability monitoring.

For membrane integrity verification, we incubated Vero cells with 5 μM CMFDA (5-chloromethylfluorescein diacetate; Invitrogen) for 30 min at 37°C in serum-free RPMI. CMFDA is cell membrane permeable and nonfluorescent. When internalized, it is cleaved by esterases present in the cytoplasm, becoming fluorescent and impermeable (16). If membrane integrity were lost before parasites egress, extravasation or decreased intracellular fluorescence would occur. Subsequently cells were washed twice and incubated with RPMI–10% FBS. Additionally, we incubated the cells with 10 μg/ml propidium iodide (Invitrogen), a compound fluorescent when intercalated with DNA molecules and permeable only to cells with compromised cytoplasmic membrane. Cell monitoring was performed by confocal microscopy (TCS SP5 II tandem scanner; Leica) in time-lapse assays.

### Confocal microscopy and live-cell imaging.

Vero cells (4.5 × 10^3^) were plated in a μ-Dish (35-mm, high Grid-500; ibidi) and infected with trypomastigotes (multiplicity of infection [MOI] of 100:1). Time-lapse acquisition was performed under physiological conditions (humidified atmosphere at 37°C and 5% CO_2_) in a TCS SP5 II tandem scanner (Leica) confocal microscope with a 63× NA 1.40 PlanApo oil immersion objective. Image processing, analysis, and multidimensional reconstructions were performed with Imaris 7.0 software (Bitplane) and ImageJ (NIH).

### Scanning electron microscopy.

Samples were fixed with 2.5% glutaraldehyde in 0.1M sodium cacodylate buffer (pH 7.2). After impregnation with 1% osmium tetroxide, samples were dehydrated with ethanol gradient solutions and dried by the critical point protocol from CO_2_. Finally, coverslips were glued in stubs sputter coated with gold. Cells were observed and documented in a FEI Quanta FEG 250 scanning electron microscope at the Electron Microscopy Center, Escola Paulista de Medicina-Universidade Federal de São Paulo (EPM-UNIFESP).

### Correlative microscopy.

Correlative microscopy was generated first by confocal microscopy observations: live-cell imaging or fixed cells and later preparation for scanning electron microscopy. In order to visualize the same cell in both microscopy systems, we performed our assays using the μ-Dish (35-mm, high Grid-500; Ibidi), which contains a numeric coordinated grid permitting precise tracking of cell location.

### Host cell membrane extraction and preservation of cytoskeleton filaments.

To visualize cell cytoskeleton by SEM membrane extraction, live cells were washed with 0.1 M phosphate-buffered saline (PBS; 37°C) one time to remove culture medium and treated with a membrane extraction solution containing 1% Triton X-100, 100 mM PIPES [piperazine-*N*,*N*′-bis(2-ethanesulfonic acid; pH 7.2), 4% sucrose, 1 mM MgCl_2_, 10 μM paclitaxel (Thermo), and 10 μM phalloidin (Sigma-Aldrich) (to stabilize microtubules and microfilaments, respectively) (35) for 10 min with gentle rocking at room temperature. Then, the samples were washed twice for 10 min in the same solution without detergent and fixed with 2.5% glutaraldehyde for scanning electron microscopy preparation.

### F-actin evaluation over time.

Vero cells (5 × 10^3^) were plated in 24-well plates containing coverslips, divided as follows: day 0 was without infection, and days 1 through 9 were infections at the same time with trypomastigotes (MOI of 100:1). After 24 h, the supernatant was washed three times with RPMI and replaced with new RPMI–10% FBS to remove parasites that were not internalized, providing some degree of synchrony to the intracellular parasite cycle. From infection to egress, daily the coverslips were fixed with 4% paraformaldehyde solution in PBS for 20 min and then washed with PBS. The remaining coverslips were also washed daily to remove supernatant parasites, avoiding reinfection. Finally, they were all incubated with phalloidin (actin filament marker) conjugated to TRITC and DAPI (4′,6-diamidino-2-phenylindole) for nuclei and kinetoplast staining. For verification and quantification of the actin cytoskeleton pattern during infection, two independent experiments were performed and three coverslips per group were counted, with 100 cells per coverslip. Measurements of phalloidin-TRITC fluorescence signal intensity were performed using tridimensional images, acquired by confocal microscopy (Z series) reconstructed by Z project tool on ImageJ. Subsequently, using the Measurement tool, three different regions of each cell cytoplasm were selected and fluorescence intensity measured.

### Western blotting evaluation of host cell actin.

HeLa cells were plated in a 6-well plate. After 24 h, cells were infected with Y strain trypomastigotes (MOI of 100:1) overnight. Parasites were then washed, and HeLa cells not infected and infected (days 3, 5, and 7 of infection) were trypsinized and counted. Exactly counted (5 × 10^4^ cells) HeLa cell lysates (radioimmunoprecipitation assay [RIPA] buffer: 10 mM Tris-HCl [pH 8.0], 1 mM EDTA, 0.5 mM EGTA, 1% Triton X-100, 0.1% sodium deoxycholate, 0.1% SDS, 140 mM NaCl) were loaded per well in 10% SDS-PAGE, and protein expression was evaluated by Western blotting using antiactin at 1:10,000 (Cell Signaling catalog no. 3700), incubated overnight at 4°C. Coomassie stain was used to confirm equivalent loadings of cell lysate. Secondary antibodies (Sigma-Aldrich) were incubated for 1 h at room temperature at a dilution of 1:10,000. All antibody solutions and blocking steps were carried out in Tris-buffered saline (TBS–0.1% Tween 20–5% bovine serum albumin (Sigma-Aldrich). Bound antibody signals were amplified with the ECL enhanced chemiluminescence agent (GE Healthcare), and luminescent bands were visualized in an Alliance 2.7 photo documenter (UVItec).

### Anti β-actin immunofluorescence assay.

Vero or HeLa cells (5 × 10^3^) were plated in 24-well plates containing coverslips, divided as follows: day 0 represented cells without infection, and days 3 (predominantly with intracellular amastigotes) and 7 (predominantly with intracellular trypomastigotes) represented cells infected at the same time with trypomastigotes (MOI of 100:1). After 24 h, the supernatant was washed three times with RPMI and replaced with new RPMI–10% FBS to remove parasites that were not internalized. Coverslips were then washed in PBS and incubated for 10 min with pure cold acetone for fixation (since for fixation, acetone produces the best results with respect to preservation of actin for anti-β-actin antibodies) (36). Coverslips were then incubated with anti-β-actin (Sigma-Aldrich, catalog no. A5441) diluted 1:100 in blocking solution PGN (0.2% gelatin, 0.1% NaN_3_, diluted in PBS) for 1 h, subsequently reacted with Alexa Fluor 488-conjugated anti-mouse IgG at 1:200 (Invitrogen) as the secondary antibody. DAPI and phalloidin-TRITC were used to stain nuclei, kinetoplasts, and actin microfilaments. Images were acquired by a TCS SP5 II tandem scanner (Leica) confocal microscope with a 100× NA 1.44 PlanApo oil immersion objective. Image processing, analysis, and multidimensional reconstructions were performed with Imaris 7.0 software (Bitplane).

### Actin degradation assay.

A total of 8 × 10^6^ Vero cells were plated on a 15-cm plate dish, harvested by cell scraper, and resuspended in 2 ml of RPMI without serum containing 1 mM ATP and 0.2 mM MgCl_2_ to help stabilization of actin filaments (37, 38). Cells were then submitted to liquid nitrogen cryolysis by freeze-thawing for three rounds. Cell lysis was confirmed by microscope observation. Equal amounts of cell lysates corresponding to 2 × 10^5^ cells were incubated for 3 h at 37°C with amastigotes or trypomastigotes (MOI of 100:1 or 2 × 10^7^ parasites per tube), treated or not with Halt protease inhibitor cocktail following the supplier’s instructions (Thermo). Subsequently lysates were centrifuged at 4,000 × *g*, and the pellets containing parasites were incubated with sample buffer and submitted to 13% SDS-PAGE. The amount of actin was evaluated by Western blotting as described in the section “Western blotting evaluation of host cell actin,” with addition of anti-GAPDH used as loading control at 1:10,000 (Cell Signaling, catalog no. 14C10). Trypomastigotes were obtained by collection of culture medium from infected cells and amastigotes, generated as described previously (11).

### Statistical analysis.

Statistical analyses were performed with GraphPad Prism, employing one-way analysis of variance (ANOVA). Data are presented as the mean and standard deviation, where ****, indicates *P* < 0.0001 (mean significance).

10.1128/mBio.00988-21.9 VIDEO S7 Live-cell imaging of the HeLa cell until parasite egress. Download VIDEO S7, AVI file, 14.9 MB. Copyright © 2021 Ferreira et al. 2021 Ferreira et al. https://creativecommons.org/licenses/by/4.0/ This content is distributed under the terms of the Creative Commons Attribution 4.0 International license.

10.1128/mBio.00988-21.10 VIDEO S8 Egress of trypomastigotes in confluent Vero cells. Download VIDEO S8, AVI file, 15.8 MB. Copyright © 2021 Ferreira et al. 2021 Ferreira et al. https://creativecommons.org/licenses/by/4.0/ This content is distributed under the terms of the Creative Commons Attribution 4.0 International license.
