# Supplementary figures and images for: Parasite-Mediated Remodeling of the Host Microfilament Cytoskeleton Enables Rapid Egress of Trypanosoma cruzi following Membrane Rupture
Source: mBio. 2021 Jun 22;12(3):e00988-21. doi: 10.1128/mBio.00988-21 (PMC8262949; doi:10.1128/mBio.00988-21)

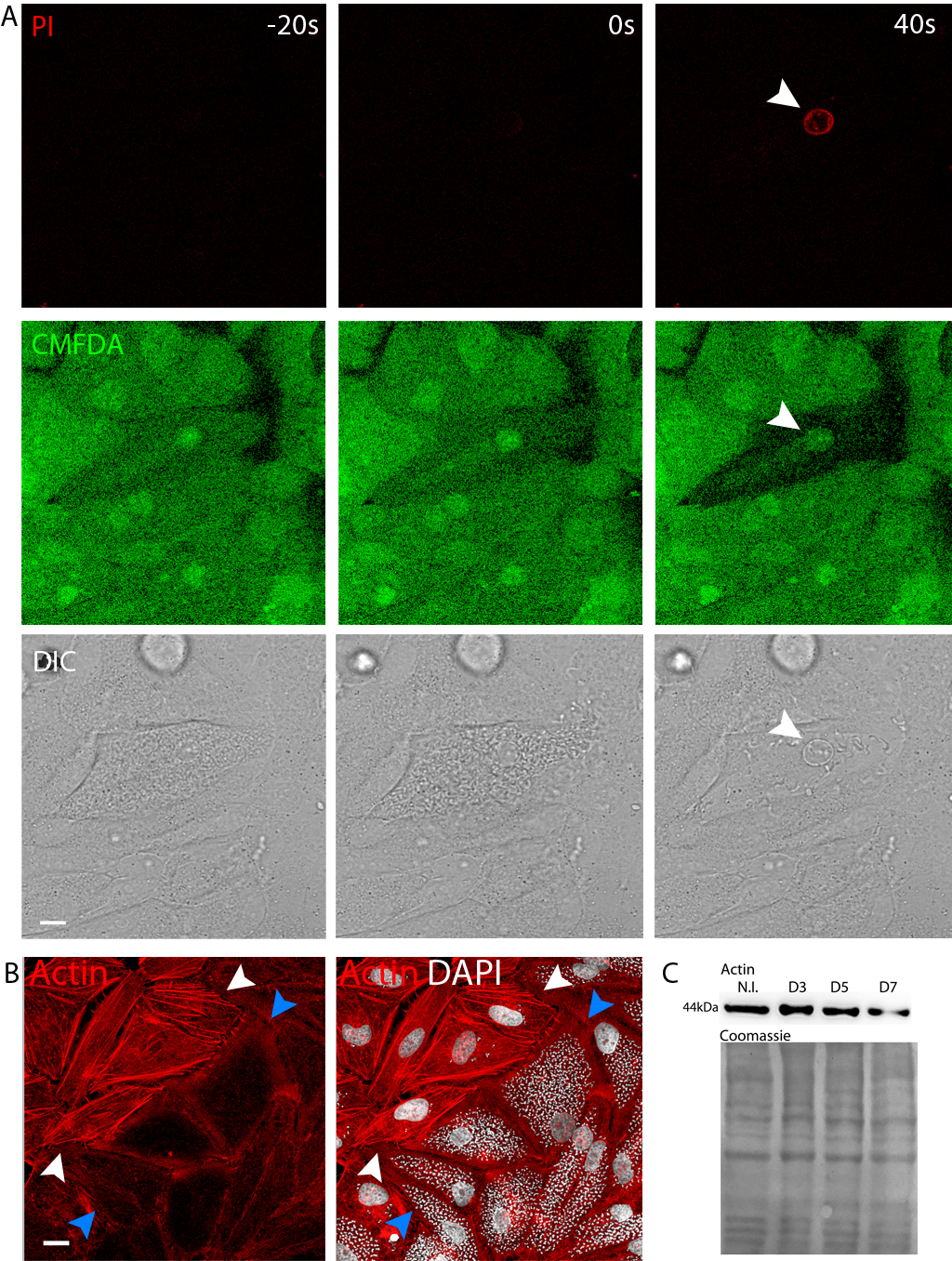

Supplement: FIG S1 [file mbio.00988-21-sf001.jpg]

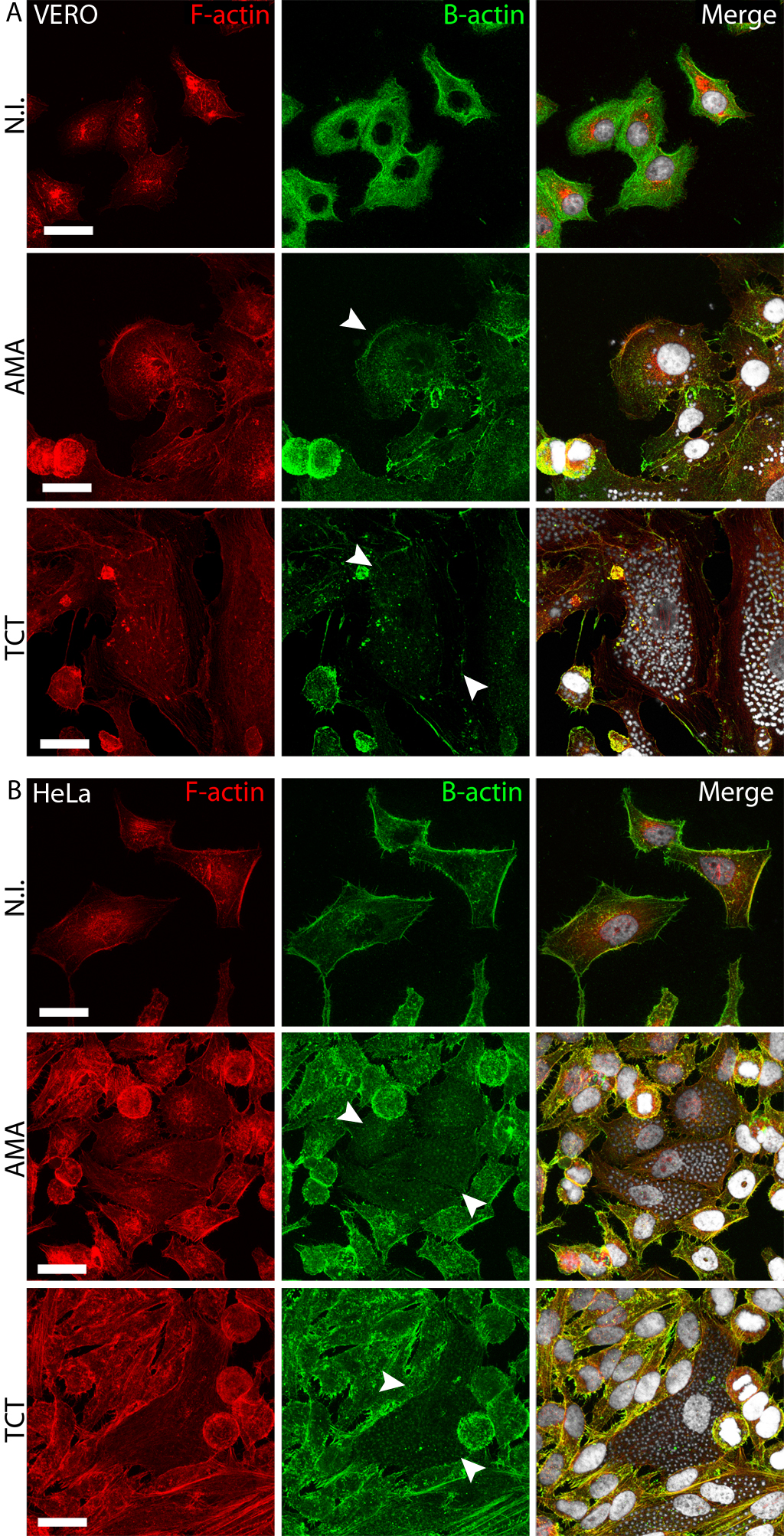

Supplement: FIG S2 [file mbio.00988-21-sf002.jpg]
